# Perceptıon scale of barrıers to contraceptıve use: a methodologıcal study

**DOI:** 10.1186/s40738-017-0038-9

**Published:** 2017-08-03

**Authors:** Selma Sen, Aynur Cetinkaya, Aysel Cavuslar

**Affiliations:** 1 Midwifery Department, Faculty of Sciences, Celal Bayar University, 45100 Sehzadeler, Manisa, Turkey; 2 Nursing Department, Faculty of Sciences, Celal Bayar University, Manisa, Turkey; 3 Hafsa Sultan Hospital, Celal Bayar University, Manisa, Turkey

**Keywords:** Scale development, Contraceptive method, Obstacles, Perception

## Abstract

**Background:**

The objective of this study was to design and develop the Perception Scale of Barriers to Contraceptive Use (PSBCU) as a measurement tool for the qualitative assessment of the barriers and obstacles women perceived with regard to contraceptive use or low rates of contraceptive use in women using family planning services.

**Method:**

The data for this methodological study were collected using the face-to-face interview technique from 320 married women between the ages of 15-49 who were attending clinics at the Hafsa Sultan Hospital, CBU. The data collection tools used in the study, which was carried out from May to September 2014, were the “Introductory Information Form” and the “Perception Scale of Barriers to Contraceptive Use”. Language validity and construct validity (explanatory factor analysis) were applied in order to test the validity of the Perception Scale of Barriers to Contraceptive Use.

**Results:**

Kaiser Meier Olkin (KMO) analysis was performed to determine the availability of the scale for the size of participants. The sample adequacy calculated as the KMO value was 0.916 and the Bartlett’s Test of Sphericity (X^2^ = 6721.793 *p* < 0.000) sample size analysis value was found to be sufficient for factor analysis. The total Cronbach’s Alpha coefficient of 34 items which included three factors explaining 54.95% of the variance after Varimax rotation was calculated to be 0.95. The largest factor was the “cognitive domain” explaining 18.89% of the variance, followed by the “emotional domain” explaining 18.05% of the variance, and finally the “social domain” explaining 18.01% of the variance. Item-total score correlation coefficients of scale items were found to be between 0.54 and 0.83.

**Conclusions:**

The study demonstrateded that the “Perception Scale of Barriers to Contraceptive Use” was valid and reliable. We believe that the scale is suitable for use by women in a family planning education and training programs in order to evaluate their situation. It should also be assessed for validity and reliability for different groups (adolescents, men, etc.).

## Background

In general terms, the use of contraceptives, which indicates how developed a country is and gives insight into people’s behaviors regarding their health, demonstrates the amount of conscious effort used by couples for the purpose of controlling their own reproduction. An increase in the use of contraception is often one of the most significant signs of decreasing reproduction in developing countries [1]. Using contraception has a determinative effect on societies, their development and the health and status of women within them. In order to meet the requirements of several Millenium Development Goals related to child mortality, maternal health, HIV/AIDS and gender equality, contraception is necessary. It functions as a major tool for assessing improvements and developments in reproductive health services. However, the WHO has found that large differences remain in different parts of the world with regard to knowledge about and education related to contraceptive methods [2].

In fact, 26.5 million of the 87 million unintentional, badly-timed or unwanted pregnancies are a result of using contraceptives in an incorrect way, or of the failure of contraceptives. Contraception’s primary contribution to the decrease of maternal mortality and disability can be attributed to its ability to lessen the number of unsafe miscarriages. Providing contraceptives for those women wishing to wait for specific periods of time before becoming pregnant again is likely to be a factor in preventing unwished for pregnancies that come with a high risk [3, 4].

Studies carried out worldwide and in Turkey demonstrate that which method is chosen to control reproduction often relates to patriarchal traditions. These can influence how women behave with regard to their reproductive rights [5]. This is because the use of contraceptive methods is one of most the significant factors affecting the level of fertility. In Turkey, an increase in the use of contraceptive methods has been seen. This is especially true with regard to modern contraceptive methods (contraceptive pill – combined and progestogen only, injection, implant, IUD/IUS, male condom, female condom, diaphragm/cap, and spermicides). Nevertheless, although extremely reliable and harmless contraceptive methods are widely available for both men and women, the use of traditional methods remains a common problem. A significant number of couples either use no methods at all or, alternatively, try to avoid pregnancy by using unreliable, inefficient and sometimes potentially harmful contraceptive methods [6].

When discussing nursing/midwifery, with is theoretically and conceptually focused on the health of patients, researchers and theorists have tended to concentrate their attention on studies about health, health behaviors and the kinds of issues which can affect patients’ health [7]. Approaches to health today often look at how individuals make decisions and take actions in order to look after, maintain and increase their well-being [8, 9]. In addition, following the motto used by nurses in Turkey, “Something for everyone, more to those in need”, the most important responsibilities of nurses/midwives working in essential health services are to look after fertile women between 15 and 49 years old, who constitute the main group at risk, to decrease infant mortality and to improve the effectiveness of family planning services. It is partiucarly important that nurses and midwives offering education, consultation and guidance about family planning and contraceptive methods, and that they help motivate their patients [10]. Nurses and midwives offering these services should understand that individuals (whether men or women) may have different intellectual capabilities and different knowledge of contraception, and they should learn about their patients’ past use of contraceptive methods [11]. Because of this, how each method works, how frequently it is found within the health service, what individuals think about it, what experiences individuals have had with it, and the obstacles they face in continuing to use it, should all be assessed.

### Contraceptive use

The choice and use of a contraceptive method is a personal matter [12]. A linear correlation has been demonstrated between the rate of contraceptive use and educational level. Among women with a high school education the rate of contraceptive use is four times higher than that of uneducated women. The effects of being able to access information and gain understanding are clearly shown by this situation. The higher the educational level, the higher a woman’s social status is likely to be and with this comes the expectation that educated women will make their own life choices [13]. A woman’s age is also a signficant factor in contraceptive use. In women aged 25-29, there has been an apparent growth in the use of contraceptives [14]. In Turkey contraceptive methods are used more in the west of the country, by those with a better education and those who have one to four living children [15].

Factors which may influence contraceptive use include its accessibility, its cost, the couple’s desire to have sexual intercourse, any limits of the methods, widespread contraceptive use, variety, degree of failure experiences, side effects, secondary benefits and the couple’s dependence on medical personnel [1, 16]. Social expectations may influence the choice and use of contraception negating specific options or promoting specific norms [16]. Contraceptive use is also affected by autonomous factors such as the presence/absence of family planning services and their quality, cultural traditions, marriage practices and traditional activities related to the interval between pregnancies [17]. A country’s laws and policies may prohibit, restrict or facilitate contraceptive use [18].

#### Previous studies on contraceptive use

In a study by Fathizadeh et al. in Iran, which deployed a phenomenological design to assess young women’s contraceptive use early in their marriage, four categories were found: impedimental factors, motivating factors, exchanging factors and abandonment factors. The authors noted that women had both positive and negative experiences [12]. A study by Sabble et al. in the USA demonstrated that visits by community health nurses reduced perceived obstacles to the use of contraceptives (accessibility, acceptance and appropriateness) and developed the self-efficacy of individuals in terms of contraceptive use [18].

In a study conducted by Aikena et al., 21.86% of women and 24.52% of men stated that they had used a contraceptive pill during their most recent intercourse and 17.24% of women and 20.43% of men stated that they had used condom [19]. A study conducted by Schwandt et al. noted that 3% of Nigerian women used injectables, 2% male condoms, 2% the oral contraceptive pill, 1% IUD [20]. A study by Medhanyie et al. found that in Northern Ethiopia 35.6% of women in total used contraceptive methods and that the rate was 41.0% among married women [21]. Sweya et al. found that 43.6% of university students stated that they had previously used contraceptives, while 40.4% were currently using contraceptivs [22].

In another study conducted with women requesting pregnancy tests, six areas which influenced the decision to use contraceptives were access, social norms, denial about pregnancy, embarrassment, forced sex and other miscellaneous concerns [23].

## Methods

The study functions as a methodological research study. The study demonstrates that we have produced a valid and reliable measurement tool for determining perceived obstacles to the use of contraceptive methods among Turkish women.

### Participants

The participants consisted of individuals receiving treatment between May and September 2014 in the gynaecology clinic of Hafsa Sultan Hospital, Celal Bayar University, Manisa. A total of 320 women were asked to participate in the study. The womenparticipating in the research were selected according to the Simple random sampling method. The criteria for selection were being female, literate, between the ages of 15-49, married, not receiving psychological treatment, being able to communicate verbally and voluntarily agreeing to participate in the study. The objective of the study was explained; an assurance was given that the answers would remain confidential and the interview setting was discussed. A total of 320 women volunteered.

### Information about the clinical setting

The study was carried out in the gynecology clinic of Hafsa Sultan Hospital, Celal Bayar University, Manisa. Approximately 150 patients are assessed or treated in this clinic daily. All the various diagnostic and treatment methods for the purposes of ensuring reproductive health are used in these polyclinics.

### Sample size

In order to calculate the sample size in scale development studies, it is frequently recommended that five to ten subjects be included per item, dependant on the number of items in the draft scale [24]. For this reason, a total of 320 cases was thought sufficient to perform reliability/validity analyses, as the scale was comprised of 31 items (31 × 10 = 310).

### Procedures for scale development and analyses

Three stages were followed in developing the scale: A literature review and deep interviews to generate the item pool, content validity testing and appplication of a pilot test.

### Literature review and deep interviews to generate item pool

In the first stage, a comprehensive review of the literature was undertaken. The researchers read articles and books containing a variety of statements about contraceptive use and statistics books about scale development in order to formulate the question pool. Some of these works have been cited in the reference section. Following the review of the literature and having received the opinions of health professionals working in the field of reproductive health, a pool was designed for the scale items. Two discrete forms were created by the researchers at the end of the literature review. Form I includes 26 descriptive questions on sociodemographic characteristics, obstetric characteristics and contraceptive use. An item pool of 48 questions was formulated for Form II which was reduced to 32 items by the researchers after expert opinions had been received in which the theoretical content of the questions was examined.

### Perception scale of barriers to contraceptive use (PSBCU)

**Fig. 1:**
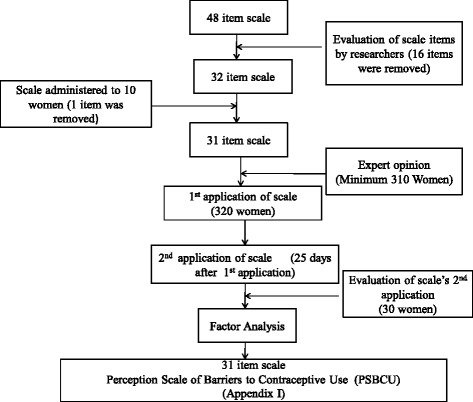
Flowchart of the stage in the development of the Perception Scale of Barriers to Contraceptive Use (PSBCU)

The Perception Scale of Barriers to Contraceptive Use (PSBCU) was designed as a 5-point Likert scale. Following a review of the literature and having received expert opionions, a pool was developed for scale items. An item pool of 48 questions was formulated which was reduced to 32 items by the researchers after experts in the field had examined the theoretical content of the scale. The scale was pilot-tested with ten women from the research population registered in the family health center who were subsequently not included in the full study, One item that these participants could not answer was deleteded from the scale as it was unsuitable. The second application was carried out 25 days after the first application and the scale then assumed its final form with 31 items after factor analysis (Fig. 1).

Each perceived obstacle was assessed by women using a 5-point Likert scale. This ranged from “Strongly Agree (5)” to “Strongly Disagree (1)”. The lowest score obtainable from the scale is 31, whereas the highest score is 155. A high score indicates a high level of perceived obstacles.

In the administration of the scale every dimension on the tool was evaluated separately and all sub-scales were added together to produce one combined score. Eleven items on the scale involve obstacles related to the “Emotional Dimension”, 11 items involve obstacles related to the “Social Dimension” and 9 items involve obstacles related to the “Cognitive Dimension”. No items are to be reverse-coded in the scale.

The necessary permissions were obtained from the Local Ethics Board of the Faculty of Medicine, Celal Bayar University and Hafsa Sultan Hospital, Celal Bayar University. After they had been informed by the researcher about the tests and the procedure, the verbal consent of the women was sought and obtained. At this stage, the researchers formulated the Perception Scale of Barriers to Contraceptive Use (PSBCU) in accordance with the literature. After the pilot test (10 women) had been performed and expert opinions (10 experts) received, the women in the sample were examined and clinical procedures applied. The women were then taken into a room to fill in the forms using the face-to-face interview technique. The aim of the study was explained to them, an assurance was given that answers would remain confidential and the interview setting was discussed.

Thirty women of the 320 volunteeredfor a re-test. It took approximately 15 min to fill in the self-report forms and the PSBCU itself took approximately 20 min to complete. Women were asked to use a pseudonym in order to match the data obtained in the first application with the data obtained in the second application.

Kaiser Meier Olkin (KMO) analysis was carried out to determine the availability of the scale for the size of participants. Validity and reliability analyses of the scale were thenperformed.

### Statistical analysis

The analysis of the data was encoded in SPSS 15 (Statistical Package for Social Sciences) and number-percentage distribution of the introductory information was carried out.

#### Validity analyses

##### Content validity of PSBCU

The scale’s content validity analysis was carried out by seeking the opinions of ten experts in community health, community health nursing, maternity health, maternity nursing and family medicine. Content Validity Index (CVI) (considering the value significant at the *p* < 0.05 significance level, i.e. 0.62 for ten experts) was used to work out the validity rates of all items [21].

##### Construct validity of PSBCU

Bartlett’s Test of Sphericity was applied to assess the suitability of the sample size for factor analysis. Explanatory factor analysis was carried out for the construct validity of the scale and relations between the results of previous studies and the current study’s results were defined [25–28].

#### Reliability analyses

A re-test was carried outed with 30 women who had volunteered for the second application. Stability over time (test-retest reliability) was assessed using the Pearson product moment correlation coefficient technique. Internal consistency was evaluateded using Cronbach alpha calculations, item-total score correlation technique, split-half and Spearman-Brown reliability coefficients [26–28].

### Data collection

The scale consisting of 31 items was applied to 320 women. They filled in the scale separately in a private room while they were waiting for to be examined. The time taken to fill in the scale was approximately 10–15 min.

### Data analysis

The items in the scale varied from 1, “Strongly disagree”, to 5, “Strongly agree”**.** The total score was calculated by adding up the score from each item. The lowest score obtainable from the scale is 31, whereas the highest score is 155. A high score indicates a high level of perceived obstacles. The data was recorded onto the database. To evaluate construct validity, the value of Kaiser-Meyer-Olkin was 0.916 and Bartlett’s X^2^ = 6721.793 (*p* < 0.000).

The Statistical Package for Social Sciences (SPSS) 15.0 for Windows was used to analyse the data. Descriptive statistics, factor analyses and the Cronbach alpha test were used. The statistical significance level for confidence was taken to be 95% and for *p* values, 0.05.

## Results

### Introductory and demographic characteristics of women

The mean age of women in the study group was 29.67 ± 6.31 (Min:20 Max:50, Median:29),. More than half of the participants (56.4%) were married and 82.3% lived in a nuclear family. The majority (40.6%) had an undergraduate degree and the average time in employment was 9 ± 7 years 9 ± 7 (Min:1- Max: 30, Median:8) and 86.1% were service nurses. 42.5% reported a high level job satisfaction; 76.3% had no membership with any professional associations.

### Validity and reliability methods

Table 1 shows items of the scale used in the study and descriptive statistics based on the data obtained. Mean, standard deviation and peak values of the participants’ answers to the 31 items on the scale were calculated. In these results, the mean scores of scale items vary from 1.92 to 2.94 (Table 1).

**Table 1:** Items in the perception scale of barriers to contraceptive use and descriptive statistics

Perception Scale of Barriers to Contraceptive Use: Any contraceptive method:	N	M	SD	Peak value
Emotional Domain	320	26.75	8.25	22
1. It is not suitable for me.	320	2.62	1.30	2
2. It may be painful.	320	2.60	1.09	2
3. It is time-consuming.	320	2.35	1.03	2
4. It prevents my daily activities.	320	2.12	1.91	2
5. It disturbs my sex life.	320	2.31	1.03	2
6. There are a lot of risks.	320	2.53	1.07	2
7. Is is too expensive.	320	2.29	0.98	2
8. I’m concerned about having a bad reaction.	320	2.55	1.11	2
9. It is scary for me.	320	2.40	1.10	2
10. It is a serious matter for me.	320	2.50	1.10	2
11. Prolonged use affects me negatively.	320	2.43	1.11	2
Social Domain	320	23.81	7.62	22
12. It affects my working life negatively.	320	2.14	0.96	2
13. It affects my husband negatively.	320	2.22	0.99	2
14. It affects attitudes of people towards me negatively.	320	1.92	0.81	2
15. It is sad for me.	320	2.03	0.84	2
16. It is sad for my husband.	320	2.11	0.94	2
17. It is time-consuming to learn about it.	320	2.18	0.91	2
18. It is time-consuming to get used to it.	320	2.30	0.95	2
19. It is time-consuming to get information.	320	2.20	0.94	2
20. It is time-consuming to go to examinations.	320	2.29	1.03	2
21. It is difficult.	320	2.31	1.03	2
22. I have to take a long break from work for it.	320	2.07	0.90	2
Cognitive Domain	320	19.86	6.60	18
23. I find it odd/strange.	320	2.23	1.01	2
24. I find it embarrassing.	320	2.08	0.90	2
25. It does not fit in with our culture.	320	2.02	0.88	2
26. It does not fit in with my beliefs.	320	2.01	0.87	2
27. It is difficult to obtain access to it.	320	2.09	0.89	2
28. It is embarrassing to obtain it.	320	2.04	0.89	2
29. It is not hygienic.	320	2.18	0.99	2
30. My husband does not want it.	320	2.23	1.08	2
31. I cannot talk to a male health professional about it.	320	2.94	1.43	2
Total	320	70.44	20.00	62

### Validity analyses of perception scale of barriers to contraceptive use (PSBCU)

For content validity, practicality and understandability the evaluation scores of each item in the PSBCU were calculated according to the expert opinion. Content Validity Index (CVI) (considering the value significant at the *p* < 0.05 significance level, i.e. 0.62 for ten experts) was used to work out the validity rate of all the items, which was 0.74 [29].

Explanatory factor analysis was deployed to evaluate the construct validity of the scale. Factor analysis involves gathering a high number of variables under a small number of headings [27, 28]. In this study, factors with an eigenvalue greater than 1 were included in the assessment, which is known as the Kaiser-Guttman rule.

The sample adequacy calculated as the KMO value was 0.916 and the Bartlett’s Test of Sphericity (X^2^ = 6721.793 *p* < 0.000) sample size analysis value was found to be sufficient for factor analysis. During examination of the factor structure of the scale, scale items were released and principal components analysis led to the production of six factors with an eigenvalue above 1. However, when the factor analysis was limited to three, the values shown in Table 2 were obtained. The largest factor found after Varimax rotation was the first factor, explaining 18.89% of the variance. The second factor explained 18.05% of the variance. The third factor explained 18.05% of the variance. Thus, three factors explained 54.95% of the total variance. The first factor was named “Cognitive Domain”, the second factor was named “Emotional Domain” and the third factor was named “Social Domain”. Cronbach’s Alpha coefficient of the factors was determined to be as follows: 1st factor, Cognitive Domain: α = 0.88; 2nd factor, Emotional Domain: α = 0.89; 3rd factor, Social Domain: α = 0.91. The Cronbach’s Alpha coefficient of the whole scale was 0.95.

**Table 2:** Perception scale of barriers to contraceptive use: factor analysis

Items	Factor 1 (Cognitive Domain)	Factor 2 (Emotional Domain)	Factor 3 (Social Domain)
Item 1		0.555	
Item 2		0.577	
Item 3		0.513	
Item 4		0.677	
Item 5		0.695	
Item 6		0.761	
Item 7		0.623	
Item 8		0.666	
Item 9		0.542	
Item 10		0.570	
Item 11		0.584	
Item 12			0.555
Item 13			0.541
Item 14			0.532
Item 15			0.708
Item 16			0.723
Item 17			0.612
Item 18			0.625
Item 19			0.614
Item 20			0.518
Item 21			0.474
Item 22			0.511
Item 23	0.723		
Item 24	0.805		
Item 25	0.836		
Item 26	0.783		
Item 27	0.519		
Item 28	0.631		
Item 29	0.596		
Item 30	0.629		
Item 31	0.427		
Eigenvalues	13.187	2.431	1.418
explained variance	18.890	18.050	18.013
Sub scales’ Cronbach’s alpha coefficients	0.88	0.89	0.91
Scale’s Cronbach’s alpha coefficient	0.95		

### Reliability analyses of perception scale of barriers to contraceptive use (PSBCU)

The item analysis aimed at determining the internal consistency of the scale was carried out with 320 women. As a result of the item analysis carried out in order to determine the internal consistency of the scale, item-total score correlation coefficients of scale items was determined to be between 0.54 and 0.83. Items with a low reliability coefficient were deleted and the Cronbach’s Alpha coefficient calculation was repeated and no significant difference was seen. For this reason, all items in the scale were kept.

Another method used to assess the internal consistency reliability coefficient is to calculate ‘the half-test reliability’. To calculate half-test reliability, scale items are split into two parts of equal size and the correlation between measurement results is calculated [23]. The correlation coefficient between the Perception Scale of Barriers to Contraceptive Use (PSBCU)‘s two halves was 0.74. According to the half-test reliability results of the scale, the Cronbach’s Alpha coefficient of the first half (16 items) was 0.91, whereas the Cronbach’s Alpha coefficient of the second half was 0.92, the Guttman Split-Half coefficient was 0.89 and Spearman-Brown coefficient was 0.89. These results demonstrate that the scale has internal consistency and is reliable. The notion of whether items in the scale are equal to each other indicates that questions are answered by the participants using the same approach and that questions have similar difficulty levels [23]. These results demonstrate that women did not approach or perceivescale items in the same way and that they answered items by directly giving their opinions related to each item (Hotelling T^2^ test = 241.5380, F = 11.9952, *p* < 0.001).

After assessing the relationship between item scores in each sub-scale and the total score of the sub-scale with correlation analysis, the reliability coefficient was determined to be between *r* = 0.54 and *r* = 0.78 in the Emotional Domain and a highly significant correlation was determined for each item (*p* < 0.001). The reliability coefficient was found to be between *r* = 0.66 and *r* = 0.78 in the Social Domain and a highly significant correlation was found for each item (*p* < 0.001). The reliability coefficient was found to be between *r* = 0.59 and *r* = 0.83 in the Social Domain and a highly significant correlation was found for each item (*p* < 0.001, Table 3). After the re-test was performed with 30 women who had volunteered for the second application the stability over time (test-retest reliability) reliability coefficient was found to be *r* = 0.96.

**Table 3:** Perception Scale of Barriers to Contraceptive Use (PSBCU): Item-total score correlation

Items		Item-total Score Correlation	p	The item is deleted (Cronbach’s Alpha)
Emotional domain	Item 1	0.548	0.000	0.8954
	Item 2	0.693	0.000	0.8808
	Item 3	0.683	0.000	0.8812
	Item 4	0.730	0.000	0.8784
	Item 5	0.688	0.000	0.8809
	Item 6	0.764	0.000	0.8754
	Item 7	0.652	0.000	0.8831
	Item 8	0.750	0.000	0.8765
	Item 9	0.752	0.000	0.8764
	Item 10	0.691	0.000	0.8810
	Item 11	0.705	0.000	0.8800
Social domain	Item 12	0.660	0.000	0.9115
	Item 13	0.704	0.000	0.9090
	Item 14	0.686	0.000	0.9092
	Item 15	0.768	0.000	0.9049
	Item 16	0.779	0.000	0.9041
	Item 17	0.787	0.000	0.9036
	Item 18	0.775	0.000	0.9043
	Item 19	0.771	0.000	0.9046
	Item 20	0.742	0.000	0.9069
	Item 21	0.743	0.000	0.9068
	Item 22	0.679	0.000	0.9099
Cognitive domain	Item 23	0.771	0.000	0.8702
	Item 24	0.837	0.000	0.8642
	Item 25	0.814	0.000	0.8666
	Item 26	0.779	0.000	0.8699
	Item 27	0.724	0.000	0.8747
	Item 28	0.756	0.000	0.8719
	Item 29	0.732	0.000	0.8742
	Item 30	0.705	0.000	0.8781
	Item 31	0.591	0.000	0.9055
N: 320 Cronbach’s Alpha: 0.95				

Correlations between the participants’ Perception Scale of Barriers to Contraceptive Use (PSBCU) scores and sub-scales are shown in Table 4.

**Table 4:** Perception Scale of Barriers to Contraceptive Use (PSBCU)‘s first application correlations between subscales (*n* = 320)

	Emotional Domain	Social Domain	Cognitive Domain	Total
Emotional Domain	*r* = 1.000			
Social Domain	*r* = 0.719 *p* = 0.000	*r* = 1.000		
Cognitive Domain	*r* = 0.581 *p* = 0.000	*r* = 0.760 *p* = 0.000	*r* = 1.000	
TOTAL	*r* = 0.878 *p* = 0.000	*r* = 0.929 *p* = 0.000	*r* = 0.859 *p* = 0.000	*r* = 1.000

## Discussion

The main purpose of the first application in this study was to design a measurement instrument. In the development of Likert type instruments, the items’ adequacy is evaluated with superficial validity/appearance validity and practical validity studies. It has been previously found that the opinions of at least three to five experts are required to determine the validity of an instrument’s theoretical forms [25–28]. In this study, the validity rates for all items for 10 expert opinions were calculated to be above 0.62 and the content validity index for 31 items was calculated to be 0.74 (*p* < 0.05) [29]. After the pilot test, one item was deleted from the scale and the “least appropriate” items, according to the expert opinions, were reformulated.

According to Comrey and Lee [28], a sample size of 50 is very low, 100 is low, 200 is appropriate, 300 is good, 500 is very good and 1000 is excellent for scale development studies [30]. It has therefore been recommended that the sample size be at least 300 or at a minimum ratio of participants to items (5:1 or 10:1) in scale development studies [31]. In this study the same 320 participants were used for the statistical analysis that could be calculated for the first and second application, *n* = 30. The number of scale items was 31.

In carrying out factor analysis in scale development studies, the Kaiser-Meyer-Olkin (KMO) test is applied to determine the adequacy of the sample size. A scale with a KMO value between 0.70 and 0.79 is seen as good and a scale with a KMO value between 0.60 and 0.69 is seen as moderate. In this respect, the sample adequacy calculated as the KMO value was 0.916 and the Bartlett’s Test of Sphericity (X^2^ = 6721.793 *p* < 0.000) sample size analysis value was found to be sufficient for factor analysis. The factor structure of the scale calculated using Principal Component Analysis was found to have “construct-content validity” [25, 32].

Furthermore, according to Tavşancıl [32], it is considered adequate that factors in a scale explain 40-60% of the total variance and the scale must have a Cronbach’s Alpha coefficient between 0.60 and 0.80 so that it can be deployed for research purposes. In this study, three factors explained 54.95% of the total variance. The Cronbach’s Alpha coefficient of sub-scales and the scale as a whole was high (0.95) (Tables 2 and 3). These results demonstrate that the scale has internal consistency. The items in the scale cohere with each other and assess elements of the same feature and the scale’s homogeneity of the scale is adequate.

The correlation coefficient between the Perception Scale of Barriers to Contraceptive Use (PSBCU)‘s two halves was 0.74. According to the half-test reliability results of the scale, the Cronbach’s Alpha coefficient of the first half (16 items) was 0.91, whereas the Cronbach’s Alpha coefficient of the second half was 0.92, the Guttman Split-Half coefficient was 0.89 and the Spearman-Brown coefficient was 0.89. These results show that the scale has internal consistency and is reliable.

When the relationship between item scores under each sub-scale and the total score of the sub-scale with correlation analysis was assessed, the reliability coefficient was determined to be between *r* = 0.54 and *r* = 0.82 and a highly significant correlation was found for each item (*p* < 0.001, Table 3). A re-test was carried out with 30 women who had volunteered for the second application and the stability over time (test-retest reliability) reliability coefficient was found to be *r* = 96. These results demonstrate that the instrument has an adequate level of consistency over time.

## Conclusions

At the end of the factor analysis of the 31-item PSBCU, three sub-scales explaining 54.95% of the total variance were found. These sub-scales determine obstacles perceived by women in relation to contraceptive use as being in the “Emotional Domain”, “Social Domain” and “Cognitive Domain”.

As a result of the analyses, the total Cronbach’s Alpha coefficient of the 31-item PSBCU was determined to be 0.95 and the scale was found to be valid and reliable for Turkish women. In terms of sub-scales, it was found that “Emotional Domain”, “Social Domain” and “Cognitive Domain” had a Cronbach’s Alpha coefficient between 0.88 and 0.91 and were reliable. The lowest total score obtainable from the PSBCU is 31, whereas the highest score is 155. A high score indicates a high degree of perceived obstacles for women with regard to contraceptive use and shows that they have negative opinions about using of contraception.

It would be useful to test the scale further by making repeated measurements for variables which could affect contraceptive use (age, gender, educational level, social status, experiences, ethnic group, etc.) and the scale could be applied to groups that are larger and that have different characteristics to obtain further evidence for its construct validity. In addition, the socio-demographic characteristics of the women making up the sample could be regarded as a limitation of the study. The final weakness of the study is that the scale is in Turkish. For other societies, linguistic and cultural validity analysis will have to be carried out before it is applied. Nevertheless, the PSBCU scale is a simple tool that is suitable for use by all health professionals who provide family planning services.

In conclusion, we believe that the scale will be of use for health professionals working with women to prevent unwanted pregnancies in terms of determining the obstacles they face, and that it will guide health professionals when determining fertile women’s attitudes toward contraception and planning educational initiatives in relation to the use of contraceptives.
